# Attenuation of *Plasmodium falciparum* in vitro drug resistance phenotype following culture adaptation compared to fresh clinical isolates in Cambodia

**DOI:** 10.1186/s12936-015-1021-8

**Published:** 2015-12-02

**Authors:** Suwanna Chaorattanakawee, Charlotte A. Lanteri, Siratchana Sundrakes, Kritsanai Yingyuen, Panita Gosi, Nitima Chanarat, Saowaluk Wongarunkochakorn, Nillawan Buathong, Soklyda Chann, Worachet Kuntawunginn, Montri Arsanok, Jessica T. Lin, Jonathan J. Juliano, Stuart D. Tyner, Mengchuor Char, Chanthap Lon, David L. Saunders

**Affiliations:** Department of Immunology and Medicine, Armed Forces Research Institute of Medical Science, Bangkok, Thailand; Division of Infectious Diseases, School of Medicine, University of North Carolina, Chapel Hill, NC USA; National Centre for Parasitology, Entomology and Malaria Control, Phnom Penh, Cambodia; US Army Institute of Surgical Research, Joint Base San Antonio-Fort Sam Houston, San Antonio, TX USA; Microbiology Section, Department of Pathology and Area Laboratory Services, Brooke Army Medical Center, San Antonio, TX USA

**Keywords:** Malaria, In vitro drug susceptibility, Culture adaptation, Cambodia

## Abstract

**Background:**

There is currently no standardized approach for assessing in vitro anti-malarial drug susceptibility. Potential alterations in drug susceptibility results between fresh immediate ex vivo (IEV) and cryopreserved culture-adapted (CCA) *Plasmodium falciparum* isolates, as well as changes in parasite genotype during culture adaptation were investigated.

**Methods:**

The 50 % inhibitory concentration (IC_50_) of 12 *P. falciparum* isolates from Cambodia against a panel of commonly used drugs were compared using both IEV and CCA. Results were compared using both histidine-rich protein-2 ELISA (HRP-2) and SYBR-Green I fluorescence methods. Molecular genotyping and amplicon deep sequencing were also used to compare multiplicity of infection and genetic polymophisms in fresh versus culture-adapted isolates.

**Results:**

IC_50_ for culture-adapted specimens were significantly lower compared to the original fresh isolates for both HRP-2 and SYBR-Green I assays, with greater than a 50 % decline for the majority of drug-assay combinations. There were correlations between IC_50_s from IEV and CCA for most drugs assays. Infections were nearly all monoclonal, with little or no change in merozoite surface protein 1 (MSP1), MSP2, glutamate-rich protein (GLURP) or apical membrane antigen 1 (AMA1) polymorphisms, nor differences in *P. falciparum* multidrug resistance 1 gene (PfMDR1) copy number or single nucleotide polymorphisms following culture adaptation.

**Conclusions:**

The overall IC_50_ reduction combined with the correlation between fresh isolates and culture-adapted drug susceptibility assays suggests the utility of both approaches, as long as there is consistency of method, and remaining mindful of possible attenuation of resistance phenotype occurring in culture. Further study should be done in higher transmission settings where polyclonal infections are prevalent.

**Electronic supplementary material:**

The online version of this article (doi:10.1186/s12936-015-1021-8) contains supplementary material, which is available to authorized users.

## Background

In vitro drug susceptibility testing is widely used as a tool for surveillance of drug-resistant *Plasmodium falciparum.* There are two commonly used approaches to examine parasite in vitro drug susceptibility: assessing parasites isolated directly from patients (sometimes referred to as ‘immediate ex vivo’ or IEV), and from cryopreserved isolates that are subsequently culture-adapted. Culture adaptation offers convenience in sample collection and transport to a reference laboratory, particularly where there are limited field facilities to perform ex vivo assays on fresh isolates. However, the culture adaptation process at the reference laboratory is more labour intensive compared to the immediate ex vivo method. Further, recovery from cryopreservation can be a significant problem, thereby lowering the yield of the assay. While it has been argued that culture-adapted isolates represent the intrinsic drug susceptibility of parasites, regardless of host immunity and pharmacokinetics, it remains unclear whether the isolates retain their susceptibility profiles following adaptation, or whether some resistance characteristic may be attenuated in culture [1].

IEV assays on parasites isolated directly from patients are favoured by others, who argue that parasites adapted in culture are attenuated and therefore may not retain the genotype/phenotype of the original clinical isolates. This is particularly true for those genes which the parasite may have had to modify to survive in the presence of drug, and which may carry a fitness cost for the parasite. Several studies on parasite population dynamics demonstrated clonal fluctuations and selection of resistant genotypes during malaria treatment [2–4] with evidence of loss of parasite population characteristics during culture adaptation [5–7].

There is currently no standardized approach for assessing malaria drug susceptibility. In most cases, assays used depend on availability of field laboratory facilities and laboratory proximity to the collection sites. Ex vivo assays on fresh parasite isolates or short-term clinical isolate cultures intended to increase parasitaemia are favoured where adequate field laboratory facilities are available, while cryopreserved samples are preferred where field capacity is limited, but there is a well-developed central reference laboratory [8–10]. In some settings where reference laboratory proximity and transport times permit, *P. falciparum* isolates are stored at 4 °C for a few days without cryopreservative solution until transported for culture adaptation and drug testing [11].

There have been limited reports comparing parasite drug susceptibility results amongst the various approaches. Only one direct head-to-head comparison of drug sensitivity results before and after culture adaptation of the same isolates showed that chloroquine (CQ)-resistant isolates can maintain their resistance phenotype during in vitro cultivation, while isolates initially sensitive to CQ may develop resistance in culture [12]. Limited data on other drugs tested were inconclusive and corresponding parasite genetic changes were not explored. The aims of the current study were to investigate potential alterations in drug susceptibility results between fresh IEV and cryopreserved culture-adapted isolates, and explore parasite population dynamics during culture adaptation. IC_50_ results from IEV and culture-adapted Cambodian isolates were compared head-to-head using histidine-rich protein-2 (HRP-2) ELISA and SYBR Green I fluorescence assays. Selected parasite antigenic and drug resistance loci of interest were compared between the original and post-culture isolates. The results illustrate the utility of both approaches, but suggest that parasite drug resistance phenotypes may be attenuated during the parasite cultivation process, highlighting limitations on results comparability between IEV and culture-adapted drug susceptibility assessments.

## Methods

### *Plasmodium falciparum* isolate collection and specimen processing

To compare drug susceptibility results arising from fresh isolates with those from culture-adapted parasites derived from cryopreserved samples, 20 *P. falciparum* isolates were obtained from volunteers with uncomplicated mono *P. falciparum* infection enrolled in the in vitro resistance surveillance study (WRAIR#1576), conducted in multiple provinces of Cambodia (Pailin, Oddar Meancheay, Kampong Speu). A different set of 29 *P. falciparum* isolates from a clinical DHA-piperaquine study (WRAIR#1877) in Oddar Meancheay [13] was used to examine the effect of white blood cells (WBC) in culture on ex vivo drug susceptibility testing. The studies were approved by the Cambodian National Ethics Committee for Health Research (NECHR), and the Walter Reed Army Institute of Research (WRAIR) Institutional Review Board. All subjects were ≥13 years old without a history of anti-malarial drug use within the previous 7 days (WRAIR#1576) or 28 days (WRAIR#1877). Diagnosis of malaria was conducted using Giemsa-stained peripheral blood smears as reported elsewhere [10]. At the time of diagnosis (before treatment), patient blood samples were collected in heparinized tubes, processed for parasite cryopreservation and tested for *ex vivo* drug susceptibility at the field laboratory within 4 h of phlebotomy. The cryopreserved samples were shipped to the main laboratory for establishing in vitro culture to test for in vitro drug susceptibility. A portion of blood samples were collected in EDTA tubes, and shipped to the main laboratory for DNA preparation and parasite molecular analysis.

### Dried drug plate coating

Dihydroartemisinin (DHA), artesunate (AS), mefloquine hydrochloride (MQ), quinine sulfate hydrate (QN), and chloroquine diphosphate (CQ) were coated onto 96-well plates using published methods [14, 15]. Threefold serial drug dilutions were performed on plates to reach final concentrations ranging from 0.027 to 20 ng/ml for DHA and AS, 0.274 to 200 ng/ml for MQ, 1.71 to 1250 ng/mL for QN, and 2.74 to 2000 ng/ml for CQ. The top row of each plate served as a drug-free control. Drug plates were dried overnight in a running biosafety cabinet and stored at 4 °C up to 8 weeks prior to use. As a quality control for dried drug plate integrity, a sub-set of plates not used in the assays was tested to ensure an acceptable range of IC_50_ values was attained against the *P*. *falciparum* W2 reference clone, as described previously [14, 15].

### *Plasmodium falciparum* culture and drug susceptibility testing

For immediate ex vivo (IEV) drug susceptibility assay, whole blood samples from malaria patients were adjusted to 1.5 % haematocrit with 0.5 % Albumax RPMI 1640 and applied to dried drug plates at the field laboratory, without prior culture adaptation or centrifugation. Samples with >0.5 % parasitaemia were first diluted to 0.2–0.5 % by adding 50 % haematocrit human O+ red blood cells (RBCs) in 10 % serum RPMI 1640 culture media, prior adjusted to 1.5 % haematocrit with 0.5 % Albumax RPMI 1640. Plates were incubated at 37 °C using a candle jar for 72 h, after which plates were frozen and shipped to the main laboratory for parasite growth assessments using HRP-2 ELISA and SYBR Green I assays following published methods [14, 16].

To compare the drug susceptibility profiles attained from fresh and culture-adapted parasites, 20 cryopreserved *P. falciparum* isolates with parasitaemia >0.5 % were chosen for recovery, in vitro cultivation and in vitro drug susceptibility testing. All isolates were collected from patients negative for pre-existing anti-malarial activity in plasma, confirming no recent anti-malarials use [10]. Cryopreserved samples in a glycerol mixed solution were recovered as previously described [17]. Continuous culture was performed using a modified Trager and Jenson method [18]. Parasites were synchronized utilizing 5 % d-sorbitol as previously described [19], and maintained for another 48 h prior to performance of drug susceptibility assays. For drug susceptibility testing, synchronized cultures with ≥90 % ring forms were diluted to 0.5 % parasitaemia with 1.5 % haematocrit in 0.5 % Albumax RPMI 1640, and transferred to dried drug-coated plates as described previously. Plates were incubated at 37 °C with 5 % CO_2_, 5 % O_2_ and 90 % N_2_ for 72 h. Parasite growth inhibition was assessed using the HRP-2 ELISA, and SYBR Green I assay following published methods [14, 16]. During 1–2 months of in vitro culture, three to seven replicate IC_50_ assays were run on culture-adapted parasites for each isolate, and average IC_50_ were compared with those obtained from ex vivo testing on fresh samples.

In order to assess the effect of WBC on ex vivo drug susceptibility testing, 29 fresh *P. falciparum* isolates with varying parasitaemia (0.07–4.12 %) were used, as described above. A portion of the blood specimen was centrifuged and after removing the buffy coat, packed RBCs were mixed with the media as described above, and applied to the drug plate. The results were compared side by side with those obtained from whole blood samples.

### Parasite molecular analysis

Genomic DNA was extracted from EDTA blood using a Qiagen DNA extraction kit (QIAGEN, USA) according to the manufacturer’s instructions. Parasite genotyping was done using both nested PCR at polymorphic regions of the merozoite surface protein 1 (MSP1) block 2, MSP 2 (block 3), and glutamate-rich protein (GLURP) R2 repeat region to evaluate for size variation and allelic-families [20, 21] and by amplicon deep sequencing of domain 2 of apical membrane antigen 1 (AMA1) as previously described [22] (see Additional file 1). TaqMan real-time PCR methods as previously described [23–26] were used to evaluate *P. falciparum* multidrug resistance gene 1 (PfMDR1) copy number and single nucleotide polymorphisms (SNPs) at PfMDR1 codon positions 86, 184, 1034, and 1042.

### Statistical analysis

Statistical analysis was performed using GraphPad Prism version 6.0 (GraphPad Software, Inc, San Diego, CA, USA). Comparison of IC_50_s attained from fresh and culture-adapted samples were made using Wilcoxon matched pair testing. Assay correlation was evaluated using Spearman’s correlation test. Wilcoxon match pair testing was also used to compare % parasitaemia at 72 h cultivation of drug assay for whole blood versus packed RBC specimens, and their IC_50_ results. Statistical significance was defined as a *P* value <0.05.

## Results

IEV drug susceptibility testing was performed on 20 fresh *P. falciparum* isolates, attaining HRP-2 IC_50_ values for all samples. However, in trying to establish long-term culture of these 20 isolates, only 12 of 20 (60 %) could be recovered from cryopreservation and maintained in vitro. There was no difference in parasitaemia among recovered (mean parasitaemia 2.4 %, range 0.8–10.6 %) and non-recovered (3.3 % mean parasitaemia, range 1–13.8 %) isolates.

**Fig. 1:**
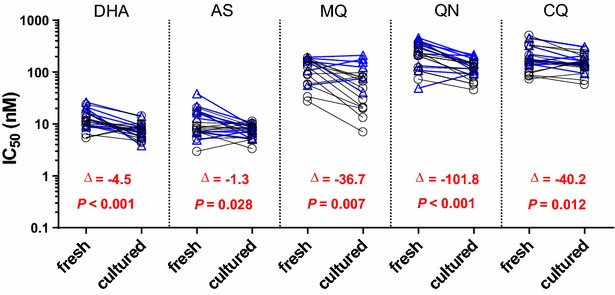
IC_50_ values were higher in 12 fresh *Plasmodium falciparum* isolates compared to when they were culture-adapted using both the HRP-2 (*black*) and SYBR Green I (*blue*) assays. Median differences and *P* values from the Wilcoxon pair test for combined HRP-2 and SYBR Green I results are indicated in *red*

**Fig. 2:**
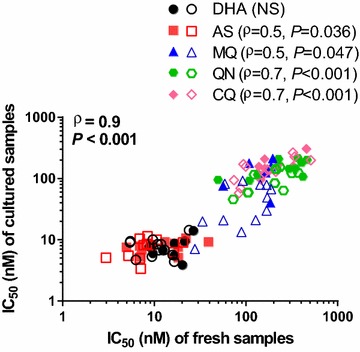
Correlations between IC_50_ values attained from paired fresh and culture adapted samples for 12 *Plasmodium falciparum* isolates using the combined values for both the HRP-2 and SYBR Green I assays. IC_50_ for each drug are presented in *different colours*, with *unfilled* and *filled symbols* representing HRP-2 and SYBR Green I IC_50_, respectively. Spearman ρ and *P* values from combined HRP-2 and SYBR Green I results are shown for each drug, with an overall Spearman’s correlation coefficient of 0.9 (*P* < 0.001)

IC_50_s obtained from culture-adapted parasites were significant lower than those from fresh isolates for all drugs tested in both the HRP-2 and SYBR Green I assays, with greater than a 50 % decline for the majority of drug-assay combinations (Fig. 1 and Additional file 2). The most dramatic decline was noted in MQ IC_50_ for nearly all isolates during in vitro culture adaptation. While consistently higher IC_50_ was found in fresh isolates, the IC_50_s of fresh and culture-adapted isolates were significantly correlated for all drugs except DHA when HRP-2 and SYBR Green I data were pooled (Fig. 2). There was a lack of correlation noted in some drugs when looking at HRP-2 results alone, and all drugs for SYBR Green I alone (Additional file 2). This is likely due to the small sample sizes involved.

To examine the possible confounding effects of human WBCs in clinical specimens, the HRP-2 assay was run on whole blood and packed RBC specimens of 29 fresh *P. falciparum* isolates, side by side. Slightly reduced IC_50_s were found after removing WBC for most of the drugs tested, except for CQ (median difference = −0.7 to −44.6, *P* < 0.05). This was not explained by effects of WBCs on parasite growth, as there were no differences in parasitaemia at 72 h in whole blood [geometric mean (range) of 0.9 % (0.1–4.6)] or packed RBC specimens [0.9 % (0.1–7.9)] (median difference of 0, *P* value 0.87).

To examine genetic constituents of original and cultured adapted parasites, three polymorphic genes (*msp1*, *msp2*, and *glurp*), *pfmdr1*drug resistance markers and a hyper-variable region in *ama1* domain II were compared between the paired original fresh and culture-adapted isolates. *Msp1, msp2*, and *glurp* genotyping indicated the same genetic pattern for original and culture adapted samples for all 12 cases. Polyclonal infections were detected in two of the 12 cases (OM144 and OM188) with all clones still present following the culture-adaptation process (Additional file 3). Similarly, the same alleles of *pfmdr1* SNPs were detected in both original and culture-adapted samples, and there were minimal changes in *pfmdr1* copy number following culture adaptation (Additional file 4). Corresponding results were also found based on AMA1 sequence analysis (Additional file 1). Deep sequencing of the hyper-variable region of AMA1 domain II detected four unique haplotypes among the 12 cases. All cases appeared monoclonal in both original and culture-adapted samples, except for case OM144. Two AMA1 clones were detected in OM144 at origin, with a minority variant occurring at 1.3 % in the fresh isolate that was lost after culture adaptation.

## Discussion

In vitro drug susceptibility results attained from fresh clinical isolates were compared and found to have reduced IC_50_ following cryopreservation and culture adaptation in an area of multidrug resistance in Cambodia. These differences were true for both HRP-2 and SYBER Green I assays, and when the results from both assays were pooled, there was good correlation between results from fresh and culture-adapted isolates for most drugs tested. Parasite molecular analysis showed no change in genetic make-up between original and culture-adapted isolates for the monoclonal infections, which predominated in this population. The overall IC_50_ reduction combined with the correlation between fresh isolates and culture-adapted drug susceptibility assays suggests the utility of both approaches, as long as there is consistency of method, and remaining mindful of possible attenuation occurring with culture. However, clonal selection during culture adaptation in polyclonal samples may occur, suggesting a limitation on performance of culture-based assays for parasite drug susceptibility of primary infections in high transmission areas where multiplicity of infection is expected to be high.

The reduced IC_50_ seen in culture-adapted samples compared to fresh isolates for the majority of commonly used drugs observed here has not been described in previous reports. A study of Kenyan isolates using the SYBR Green I assay showed no differences in IC_50_ between the original clinical isolates and culture for most drugs tested with the exception of MQ and AS which had higher IC_50_ following culture adaptation than they did originally in the IEV assay [11]. However, potential sampling bias was noted due to different collection sites in hypo-endemic and holo-endemic areas used for the culture-adapted and IEV assays. In lower transmission settings as in the present experiment, while there were clearly lower MQ IC_50_ values in cryopreserved compared to fresh isolates for the HRP-2 assay, sample size for the comparable SYBR Green I assay comparison was limited to six isolates, revealing only a non-significant trend toward higher fresh isolate values (see Additional file 2). Another study using the schizont maturation assay showed increased IC_50_ following culture adaptation, most notably for initially CQ-sensitive isolates, which became CQ-resistant during culture adaptation [12]. Although parasite genetic data were not collected, this observation was hypothesized to be related to selection of CQ-resistant minority variant clones in culture attributed to parasite metabolic changes occurring during culture adaptation [27, 28]. Given the predominance of polyclonal infections in Africa, and greater potential for differential parasite subpopulation selection during the culture adaptation process, the likelihood of extrapolating these findings to higher transmission settings remains unclear.

Little evidence of parasite sub-population selection during culture adaptation was found in this study, based on limited molecular markers analysed. Indeed, polyclonal *P. falciparum* infections are relatively uncommon in lower transmission settings such as Cambodia [20]. The considerable attenuation of IC_50_ in culture observed here cannot be explained by loss or selection of minority variants. Given that *P*. *falciparum* W2 reference was tested as a quality control for inter-assay variation, the differences in IC_50_ are unlikely due to variation among lots of drug, reagents, and media used between the in vitro and ex vivo assays. One potential explanation would be that WBCs in clinical specimens may confound results by absorbing some quantity of drug, and increase IC_50_ measured in the IEV assay. This was supported by a slight reduction in IC_50_ after removing WBCs in a separate experiment, but the effects were modest. Nonetheless, inhibitory effect of WBCs on parasite growth was not observed, and there were no differences in parasitaemia at 72 h of culture in fresh whole blood compared to packed RBC (WBC-depleted) culture. Given that this study looked at only a handful of the known molecular markers conferring anti-malarial resistance, there may be other as yet unidentified genetic and epigenetic factors, as well as changes in metabolic pathways that may explain the apparent alteration of drug susceptibility phenotypes between the original and culture-adapted isolates.

There was a modest decline in artemisinin IC_50_ following culture adaptation, more so for DHA than AS, and modest correlation in values between IEV and culture-based assessments. At the time of conducting this study, the ring-stage survival assay (RSA) for measuring artemisinin resistance in isolates was not yet available. Artemisinin (ART) susceptibility phenotypes based on % survival rate of RSA have been shown to be stage-dependent [29–31]. Very early ring stages (0–3 h after invasion) have proven to be an appropriate stage for ART susceptibility testing using the RSA (RSA^0−3 h^), as changes in % survival rate allow discrimination between ART-sensitive and resistant parasites [29]. Obtaining very early ring stages using the in vitro RSA method requires tight synchronization of the parasite culture, whereas ex vivo staging of clinical isolates (age of ring) is crucial for interpreting results from the ex vivo RSA. Even with limitation on parasite staging of ex vivo fresh isolates that may affect assay accuracy, the previous report [29] showed good correlation between in vivo parasite clearance half-life and ex vivo survival rate, and suggested ex vivo RSA may be an informative screening method for surveillance of artemisinin resistance. On the other hand, the standard IC_50_ assay requires only moderate synchronicity of >80 % ring forms, regardless of the age of the ring. This level of synchronicity can be achieved in ex vivo specimens in which only ring stages are commonly found in blood circulation with later stages tending to adhere to endothelium, and in vitro assay by simple sorbitol synchronization of parasite culture before testing. Therefore, it is unlikely that the level of synchronicity had a significant influence on the IC_50_ results, and explains the differences in IC_50_ between the in vitro and ex vivo assays.

Evidence of parasite sub-population selection in polyclonal infections during culture adaptation has been described in previous studies. Differences in *pfmdr 1* genotype pre- and post-culture have been described in polyclonal infections [24]. Loss of parasite sub-populations during culture adaptation were observed in 60–70 % of polyclonal samples collected from both Uganda and the China-Myanmar border [5, 6]. Fitness costs to resistant parasite sub-populations during in vitro culture have been postulated to alter parasite genotype [5–7]. These alterations may confound in vitro drug susceptibility results for culture-adapted parasites, obscuring potential correlations with the ex vivo assay, particularly in high transmission areas where multiplicity of infection is high [2, 20, 32, 33]. On the contrary, little effect may be expected in low transmission areas where multiplicity of infection is low, as evidenced here in Cambodian isolates [20].

## Conclusions

Study results suggest attenuation of in vitro drug resistance phenotype following culture adaptation compared to fresh clinical isolates from Cambodia. This supports the use of the IEV assay as a tool for drug resistance surveillance, saving considerable time and effort required to establish parasite cultures. In addition, the IEV approach may reduce limitations in recovery rates for cryopreserved specimens, and avoid the confounding effects of clonal selection for polyclonal infections during culture adaptation. Given limitations in field laboratory capabilities in many settings, in vitro drug susceptibility assay on culture-adapted parasite remains an informative tool, with the above-noted cautions, particularly in high transmission settings where multiplicity of infection is high. Potential variation between IEV and culture-adapted results should be considered carefully when performing inter-laboratory comparisons where different methods have been used.

## Additional files

10.1186/s12936-015-1021-8 Amplicon deep sequencing of apical membrane antigen 1.
10.1186/s12936-015-1021-8 IC_50_ obtained from 12 *Plasmodium falciparum* fresh isolates were higher than when culture-adapted from cryopreserved samples for both the HRP-2 and SYBR Green I assays.
10.1186/s12936-015-1021-8 Msp1/msp2/glurp genotypes found in original and culture-adapted samples of 12 *Plasmodium falciparum* isolates from Cambodia.
10.1186/s12936-015-1021-8 Pfmdr1 copy number and alleles found in original and culture-adapted samples of 12 *Plasmodium falciparum* isolates from Cambodia.
