# Supplementary material for: Attenuation of Plasmodium falciparum in vitro drug resistance phenotype following culture adaptation compared to fresh clinical isolates in Cambodia
Source: Malar J. 2015 Dec 2;14:486. doi: 10.1186/s12936-015-1021-8 (PMC4667454; doi:10.1186/s12936-015-1021-8)

Additional file 1

Amplicon Deep Sequencing of Apical Membrane Antigen 1 (AMA1)

A 170bp fragment corresponding to domain 2 of AMA1 was PCR amplified from both fresh and culture adapted isolates. Each sample was amplified using a sample specific forward primer (XXXXXXXXXXCCATCAGGGAAATGTCCAGT) indexed with a 10 nucleotide barcode, using the published MID sequences from Roche 454 sequencing, and a universal non-indexed reverse primer (TTTCCTGCATGTCTTGAACA). All samples were amplified and sequenced in duplicate. The PCR was carried out in a 50µl volume using 300nm of each primer, 2 units of Roche FastStart HiFidelity polymerase (Roche, Indianapolis, IN) and 2.5mM dNTP mix. Cycling conditions were 95°C for 2 minutes followed by 35 cycles of 95°C for 30 seconds, 60°C for 30 seconds and 72°C for 1 minute, followed by a final elongation for 5 minutes at 72°C. Each sample was amplified in duplicate and equimolar amounts of each barcoded PCR product were mixed. These libraries were sequenced on an Ion Torrent PGM at the UNC Microbiome Core Facility after library preparation using the Ion Plus Fragment kit (Life Technologies, Foster City, CA) and IonExpress Index Barcodes. Equimolar amounts of each library were pooled and sequenced using the 400-bp sequencing kit. Sequence reads were demultiplexed and clustered using an in house script, SeekDeep, as previously described [22]. Haplotypes were kept if they occurred in both duplicate sequencing reactions and accounted for >0.5% of the reads.

In addition, replicate control reactions, containing known concentrations of genomic DNA from 4 parasite lines from MR4 (ATCC, Manasas, VA), were also included to allow us to assess the accuracy of allele frequency estimates. This mixture contained 40% strain K1, 30% strain 7g8, 15% strain Dd2, 10% strain RO33 and 5% strain V1/S. Deep sequencing of the control mixtures returned 17,864 sequencing reads between the replicate PCRs. The estimated strain frequencies were 38% strain K1, 32% strain 7g8, 12% strain Dd2, 12 % strain R033 and 6% strain V1/S.

Replicate PCRs among the clinical samples returned and average of 10,303 reads per sample (STDEV=4455). In total four unique AMA1 haplotypes were identified, and nucleotide alignment of these four haplotype are presented below. A single AMA type was identified in all fresh isolates except OM144 which contained haplotype 1 at a frequency of 98.7% and haplotype 3 at a frequency of 1.3%. All culture adapted isolates were monoclonal by AMA1 sequencing, with sample OM144 becoming monoclonal for haplotype 1.

Nucleotide alignment of four haplotypes of Apical Membrane Antigen 1 domain 2 found in 12 *P. falciparum* isolates from Cambodia.

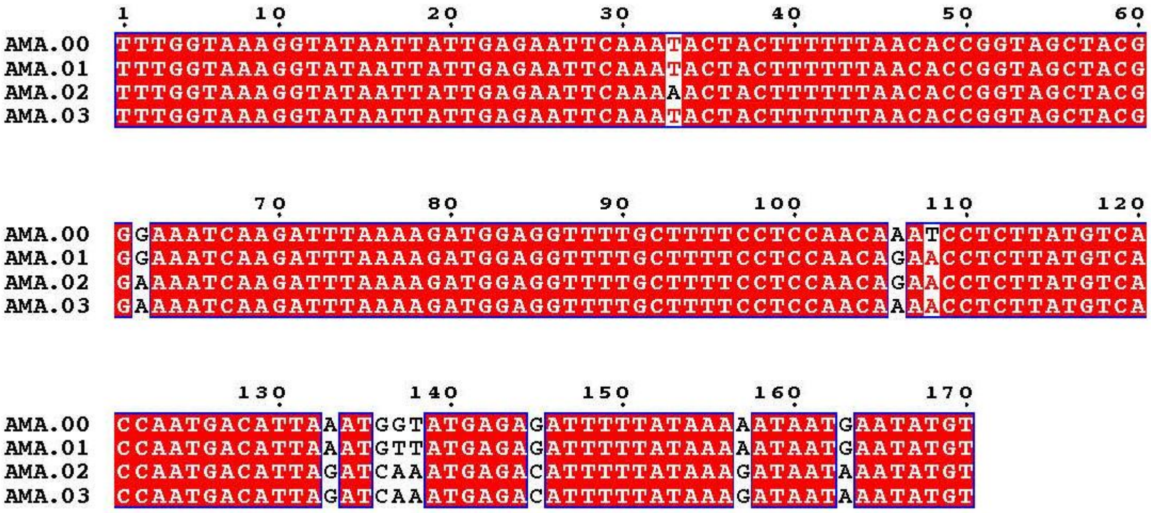

Supplement: Supplementary file 1 — 10.1186/s12936-015-1021-8 Amplicon deep sequencing of apical membrane antigen 1. [file 12936_2015_1021_MOESM1_ESM.pdf]
