# Supplementary material for: Attenuation of Plasmodium falciparum in vitro drug resistance phenotype following culture adaptation compared to fresh clinical isolates in Cambodia
Source: Malar J. 2015 Dec 2;14:486. doi: 10.1186/s12936-015-1021-8 (PMC4667454; doi:10.1186/s12936-015-1021-8)
Supplement: Supplementary file 2 — 10.1186/s12936-015-1021-8 IC50 obtained from 12 Plasmodium falciparum fresh isolates were higher than when culture-adapted from cryopreserved samples for both the HRP-2 and SYBR Green I assays. [file 12936_2015_1021_MOESM2_ESM.pdf]

Additional file 2

IC_{50} obtained from 12 *P. falciparum* fresh isolates were higher than when culture-adapted from cryopreserved samples for both the HRP-2 and SYBR Green I assays.

Geometric mean (range) IC_{50} attained from fresh and culture-adapted samples of 12 *P. falciparum* isolates are shown. HRP-2 IC_{50} were available for all 12 paired fresh/cultured samples, while only 10 isolates provided sufficient sample for SYBR Green I assay analysis. Differences in IC_{50} between fresh and culture-adapted samples were compared using the Wilcoxon pair test for median differences and *P*-values are indicated. Correlations were examined with Spearman's nonparametric correlation test (ρ) with corresponding *P*-values indicated. Significant differences and correlations are in **bold** lettering.

Assay	**GM IC_{50} (range) in nM, N (number of isolates)**				
	DHA	AS	MQ	QN	CQ
**HRP-2**					
Fresh isolates	10.6 (5.4-23.9), 12	8.2 (3.0-20.6), 12	93.2 (27.8-188.2), 12	182.3 (72.7-373.5), 12	158 (74.1-509.2), 12
Cultured isolates	7.3 (4.4-14.4), 12	7.1 (3.4-11.4), 12	35.5 (7.1-97.5), 12	92.4 (45.6-153.6),12	135.3 (57.7-264.8), 12
Wilcoxon pair test	**Δ = -3.8, *P*=0.034**	Δ = -0.1, *P*=0.519	**Δ = -70.0, *P*=0.005**	**Δ = -77.0, *P*=0.001**	Δ = -28.6, *P*=0.129
Spearman test	ρ = -0.1, *P*=0.716	ρ = 0.4 , *P*=0.169	ρ = 0.5, *P*=0.128	ρ **= 0.7 , *P*=0.019**	ρ **= 0.7, *P*=0.010**
**SYBR Green I**					
Fresh isolates	14.7 (8.8-26.6), 9	12.7 (4.9-38.9), 10	137.2 (56.2-195.5), 6	232.9 (49.5-462.1), 10	217.9 (137.6-455.3), 10
Cultured isolates	7.4 (3.9-14.1), 9	7.6 (5.1-10.7), 10	115.5 (40.7-213.5), 6	144.2 (92.8-217.5), 10	170.5 (95.2-307.9), 10
Wilcoxon pair test	**Δ = -7.8, *P*=0.004**	**Δ = -5.8, *P*=0.027**	Δ = -5.5, *P*=0.688	**Δ = -150.3, *P*=0.014**	**Δ = -47.5, *P*=0.037**
Spearman test	ρ = 0.4, *P*=0.359	ρ = 0.4, *P*=0.279	ρ = 0.4, *P*=0.419	ρ = 0.5, *P*=0.123	ρ = 0.6, *P*=0.067
