# Supplementary material for: Attenuation of Plasmodium falciparum in vitro drug resistance phenotype following culture adaptation compared to fresh clinical isolates in Cambodia
Source: Malar J. 2015 Dec 2;14:486. doi: 10.1186/s12936-015-1021-8 (PMC4667454; doi:10.1186/s12936-015-1021-8)

Additional file 3

Msp1/msp2/glurp genotypes found in original and culture adapted samples of 12 *P. falciparum* isolates from Cambodia.

Size of bands (in base pairs) are indicated on the Y-axis. Blue, pink and gray bars represent PCR bands detected for *msp1*, *msp2*, and *glurp*, respectively. Patterns of bars indicate *msp1* and *msp2* sub-families (see legend). * Denotes polyclonal infection in both original and culture adapted samples as evidenced by the presence of two *msp2* alleles.

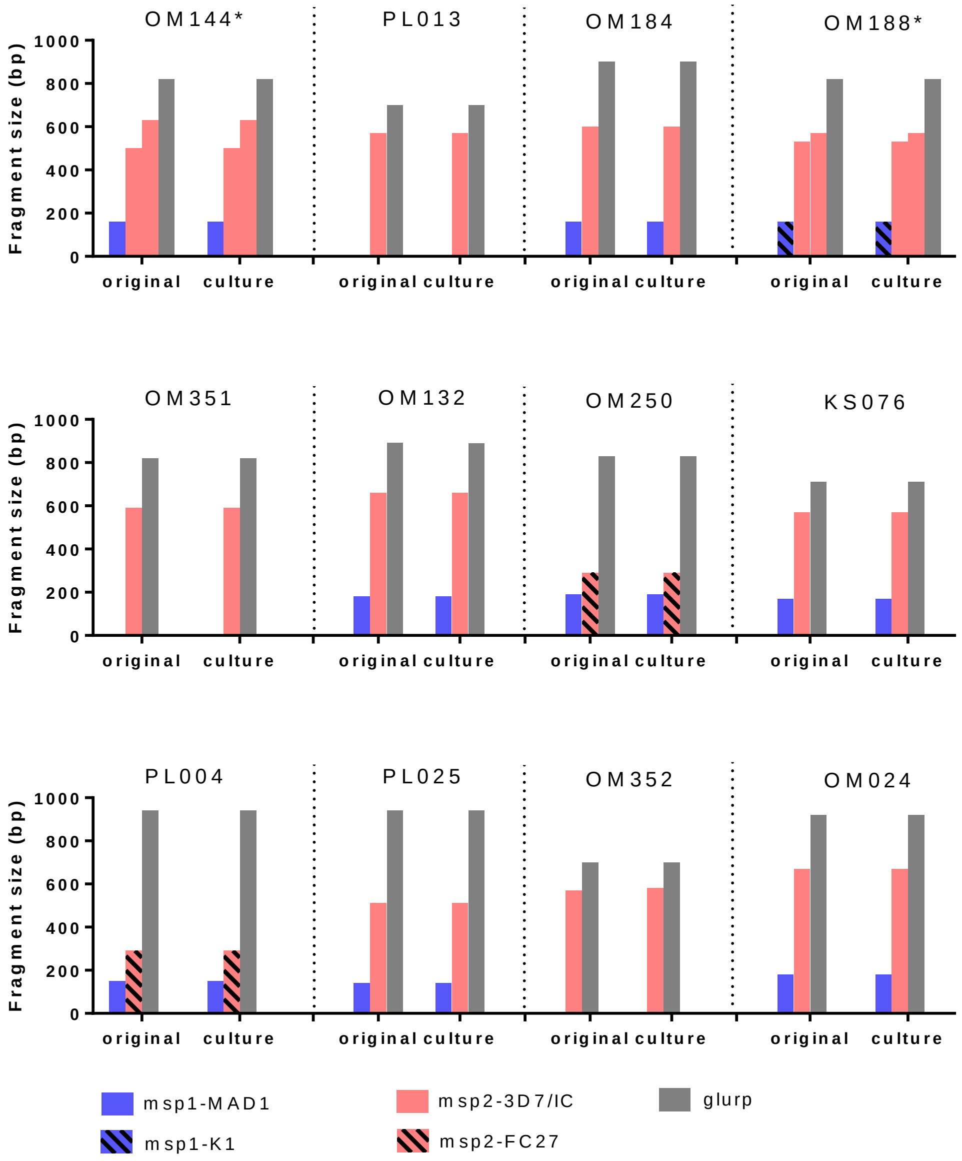

Supplement: Supplementary file 3 — 10.1186/s12936-015-1021-8 Msp1/msp2/glurp genotypes found in original and culture-adapted samples of 12 Plasmodium falciparum isolates from Cambodia. [file 12936_2015_1021_MOESM3_ESM.pdf]
