# Supplementary material for: Attenuation of Plasmodium falciparum in vitro drug resistance phenotype following culture adaptation compared to fresh clinical isolates in Cambodia
Source: Malar J. 2015 Dec 2;14:486. doi: 10.1186/s12936-015-1021-8 (PMC4667454; doi:10.1186/s12936-015-1021-8)
Supplement: Supplementary file 4 — 10.1186/s12936-015-1021-8 Pfmdr1 copy number and alleles found in original and culture-adapted samples of 12 Plasmodium falciparum isolates from Cambodia. [file 12936_2015_1021_MOESM4_ESM.pdf]

Additional file 4

Pfmdr1 copy number and alleles found in original and culture-adapted samples of 12 *P. falciparum* isolates from Cambodia.

P. falciparum isolates	Pfmdr1					
	copy number	difference in copy number	N86Y	Y184F	S1034C	N1042D
OM-144_original	1.19	0.44	N	YF	S	N
OM144_culture	1.63		N	YF	S	N
PL013_original	1.94	-0.17	N	F	S	N
PL013_culture	1.77		N	F	S	N
OM-184_original	3.08	0.07	N	F	S	N
OM184_culture	3.15		N	F	S	N
OM-188_original	1.90	0.20	N	F	S	N
OM188_culture	2.1		N	F	S	N
OM-351_original	0.99	-0.29	N	F	S	N
OM-351_culture	0.70		N	F	S	N
OM-132_original	3.30	-0.17	N	F	S	N
OM-132_culture	3.13		N	F	S	N
OM-250_original	0.80	-0.07	N	F	S	N
OM-250_culture	0.73		N	F	S	N
KS-076_original	3.40	0.37	N	F	S	N
KS-076_culture	3.77		N	F	S	N
PL-004_original	1.00	0.14	N	F	S	N
PL-004_culture	1.14		N	F	S	N
PL-025_original	0.91	0.03	N	F	S	N
PL-025_culture	0.94		N	F	S	N
OM-352_original	3.52	0.27	N	F	S	N
OM-352_culture	3.79		N	F	S	N
OM-024_original	1.72	0.39	N	F	S	N
OM-024_culture	2.11		N	F	S	N
